# A novel dataset of predictors of mortality for older Veterans living with type II diabetes

**DOI:** 10.1016/j.dib.2022.108005

**Published:** 2022-03-01

**Authors:** Avi U. Vaidya, Gabriel A. Benavidez, Julia C. Prentice, David C. Mohr, Paul R. Conlin, Kevin N. Griffith

**Affiliations:** a Department of Health Policy, Vanderbilt University Medical Center, Nashville, TN, USA; b Department of Epidemiology and Biostatistics, University of South Carolina, Columbia, SC, USA; c Department of Psychiatry, Boston University School of Medicine, Boston, MA, USA; d Center for Healthcare Organization and Implementation Research, VA Boston Healthcare System, Boston, MA, USA; e Department of Health Law, Policy and Management, Boston University School of Public Health, Boston, MA, USA; f VA Boston Healthcare System, Boston, MA, USA; g Harvard Medical School, Boston, MA, USA; h Partnered Evidence-Based Policy Resource Center, VA Boston Healthcare System, Boston, MA, USA

**Keywords:** Diabetes, Veterans, Medical care, Veterans Health Administration, Mortality prediction

## Abstract

The dataset summarized in this article includes a nationwide prevalence sample of U.S. military Veterans who were aged 65 years or older, dually enrolled in the Veterans Health Administration and traditional Medicare and had a previous diagnosis of diabetes (diabetes mellitus) as of December 2005 (*N* = 275,190) [1]. Our data were originally used to develop and validate prognostic indices of 5- and 10-year mortality among older Veterans with diabetes. We include various potential predictors including demographics (e.g., sex, age, marital status, and VA priority group), healthcare utilization (e.g., # of outpatient visits, # days of inpatient stays), medication history, and major comorbidities. This novel dataset provides researchers with an opportunity to study the associations between a large variety of individual-level risk factors and longevity for patients living with diabetes.

## Specifications Table

**Table:** 

Subject	Endocrinology, diabetes and metabolism
Specific subject area	Potential demographic and clinical predictors of mortality for elderly veterans living with diabetes
Type of data	Pre-processed data files R statistical code SQL scripts Tables Figures
How data were acquired	Data on Veterans’ demographics, clinical characteristics, medication history, prior health services utilization, and dates of death were obtained by querying the VHA Corporate Data Warehouse (CDW).
Data format	Pre-processed
Parameters for data collection	We identified a prevalence sample of Veterans with diabetes who were alive, aged ≥ 65 and enrolled in the Veterans Health Administration (VHA) as of January 1, 2006. Veterans were excluded if they were enrolled in Medicare Advantage (Part C) or did not have at least one primary care visit during 2004–2005 with records for routine biomarkers (i.e., blood pressure, body mass index, hemoglobin A1C).
Description of data collection	All data were accessed directly from the VHA Corporate Data Warehouse using SQL queries, deidentified, and then reported at the individual-level.
Data source location	VHA Corporate Data Warehouse (CDW) https://www.hsrd.research.va.gov/for_researchers/vinci/cdw.cfm
Data accessibility	Repository name: Mendeley Data Data identification number: https://data.mendeley.com/datasets/kn8v3678n9 Instructions for accessing these data: Pre-processed data files and R statistical code are publicly available for direct download.
Related research article	Griffith KN, Prentice JC, Mohr DC, & Conlin PR. (2020) Predicting 5- and 10-Year mortality risk in older adults with diabetes. *Diabetes Care* 43(8):1724–1731. doi:10.2337/dc19-1870.

## Value of the Data

• Clinical practice guidelines for diabetes treatment state that treatment goals should account for patients’ comorbidities and life expectancy. However, there are currently no nationwide, publicly available datasets of longevity for patients with diabetes (diabetes mellitus). The uncertainty of life expectancy for older adults with diabetes can make it difficult for clinicians to work with individual patients to develop ideal treatment plans.
• Our data provide a unique opportunity for researchers to estimate longevity and identify mortality risk-factors for patients living with diabetes.
• Findings may then be used to inform clinicians and patients as they participate in shared decision-making and set individualized treatment goals.

## 1 Data Description

Approximately 34.2 million Americans currently live with diabetes, of which Type II diabetes (diabetes mellitus) is the most common [2]. Diabetes is associated with increased mortality and an increased risk for other conditions including kidney disease, retinopathy, dementia, nerve damage, and high blood pressure [3]. Potential treatments include lifestyle modifications, oral antihyperglycemic medications, or insulin therapy. Clinical practice guidelines suggest that patients’ life expectancy should be taken into account when developing individualized treatment plans [4,5]. However, there are a dearth of long-term data and clinical risk prediction tools for life expectancy among patients living with diabetes.

We obtained administrative data from 2004 to 2016 for a prevalence sample of Veterans who were dually enrolled with the Veterans Health Administration and traditional Medicare, aged 65 years or older, and had a prior diagnosis of diabetes. The VHA Corporate Data Warehouse (CDW) contains data for every enrolled Veteran including outpatient and inpatient health services utilization, medication history, laboratory tests, diagnosis and procedure codes, and demographic information. Data are captured if a Veteran has an encounter at a VHA facility or with a community-based provider at VHA expense [6]. We also obtained Medicare Standard Analytic Files [7] for the same time period to obtain dates of death and to ensure we had a more comprehensive view of utilization and health conditions. The characteristics of Veterans in our dataset are presented in Table 1.

**Table 1:** Baseline characteristics of older Veterans living with diabetes (*N* = 275,190) from 2004 to 2005.

Variable	Count (%)
Age (years)	
65–69	49,501 (18.0)
70–74	85,680 (31.1)
75–79	73,389 (26.7)
80–84	50,957 (18.5)
85–89	14,230 (5.2)
90+	1433 (0.5)
Male	272,037 (98.9)
Race	
White	238,067 (86.5)
Black	28,442 (10.3)
Other	8681 (3.2)
VHA Priority Group	
1, 4 (high disability)	32,511 (11.8)
2, 3, 6 (non-compensable/low/moderate disability)	31,870 (11.6)
5 (low income)	105,228 (38.2)
7, 8 (higher income)	105,581 (38.3)
Insulin Users	42,927 (15.6)
Deaths within 5 years	65,171 (23.7)
Deaths within 10 years	157,620 (57.3)

Variable	Median ± SD

Number of outpatient visits within 2 years	30.9 ± 26.9
Number of inpatient days within 2 years	2.4 ± 18.8
Hemoglobin A1c (%)	7.1 ± 1.2
Body Mass Index (kg/m^2^)	30.0 ± 5.1
Quan-Elixhauser Comorbidity Index	4.8 ± 2.4

*Notes:* Patient baseline characteristics were determined from VA administrative data 2004–2005.

## 2 Experimental Design, Materials and Methods

**Fig. 1:**
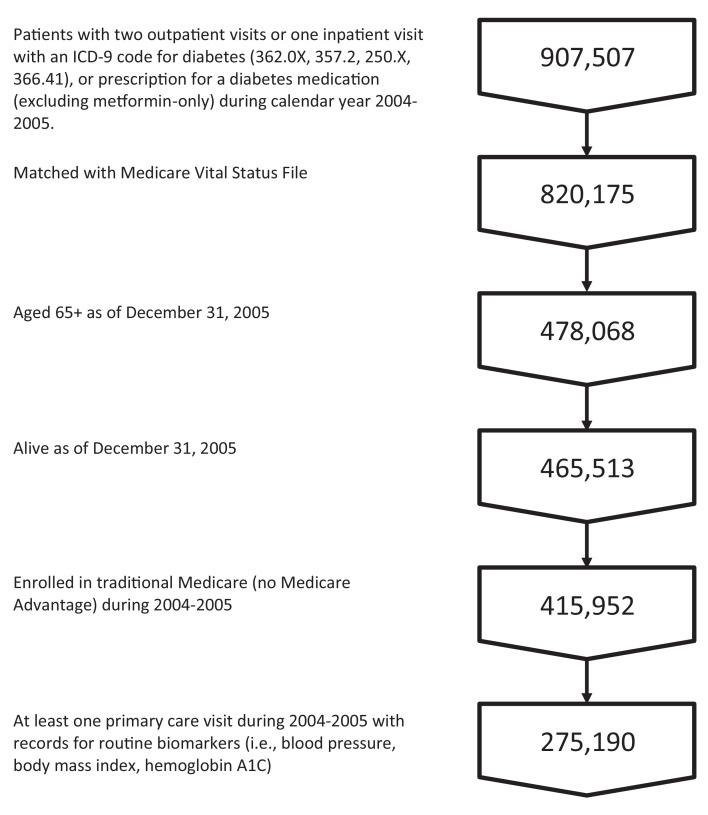
Sample selection flowchart.

We used SQL to query the VHA CDW and identify patients with either two outpatient visits or one inpatient visit with an ICD-9 code for diabetes (362.0X, 357.2, 250.X, 366.41) or a prescription for a diabetes medication (excluding metformin-only) during calendar year 2004–2005 [8]. The sample was then limited to those patients who met the following criteria; (1) alive and aged 65 or older as of December 31, 2005; (2) present in the Medicare Vital Status File (enrolled in Medicare); (3) enrolled in traditional Medicare during 2004–2005 (excluding Medicare Advantage [Part C]); and (4) were engaged in care at the VHA. This last criterion was defined as having at least one primary care visit during 2004–2005 with records for routine biomarkers (i.e., blood pressure, body mass index, hemoglobin A1C). Our final sample included 275,190 Veterans; a sample selection flowchart is presented in Fig. 1.

Predictor variables were selected based on their demonstrated associations with mortality among patients living with diabetes in previous research. Data were extracted from the following CDW tables:

• SPatient.Spatient – gender, marital status
• PatSub.PatientRace – race
• PatSub.PatientEthnicity – ethnicity
• Vital.VitalSign – mean systolic & diastolic bp (outpatient only), height, weight
• Inpatient.Inpatient/Fee.FeeInpatInvoice – count of inpatient days
• Outpat.Visit – count of outpatient visits
• RxOut.RxOutpat – medication history
• Chem.PatientLabChem – lab test orders and results
• Outpat.VDiagnosis/Inpat.InpatientDiagnosis/Fee.FeeInpatInvoice/Fee.FeeServiceProvider – International Classification of Disease 9 (ICD-9) codes
• Outpat.VProcedure/Inpat.InpatientCPTProcedure/Fee.FeeServiceProvided – Common Procedural Terminology (CPT) codes
• Patient.Enrollment – priority group, a measure of eligibility for VHA benefits based on service-related disabilities, environmental exposures, or economic hardships [9].

Veterans’ gender, age, dates of death, and enrolment in either traditional Medicare or Medicare Advantage was determined using Medicare's Master Beneficiary Summary Files. ICD-9 codes, CPT codes, counts of inpatient days, and outpatient visits were also extracted from Medicare's MedPAR and Carrier files. From the CDW, we also extracted Veterans’ priority group, demographics, ICD-9 codes, CPT codes, counts of inpatient days, and outpatient visits. Veterans’ priority group, age, sex, marital status, race, and ethnicity were retrieved as of December 31, 2005. Priority groups 1 and 4 constitute those with serious service-related disabilities (greater than 50% disability or housebound); groups 2, 3, and 6 are those with non-compensable, low, or moderate disabilities; group 5 comprises those with economic hardships; and groups 7 and 8 have no service-related disabilities and household incomes above certain thresholds. Our dataset also includes binary indicators for whether a Veteran was alive at the five-year mark (December 31, 2011), or at the ten-year mark (December 31, 2016).

We used a two-year lookback period (January 1, 2004 to December 31, 2005) to identify Veterans’ comorbidities, health services utilization, medication history, and select measures of diabetes complications. We included a variety of predictors that have been previously associated with mortality in either older adults or people living with diabetes [10]. Measures of prior health services utilization included counts of inpatient days and outpatient visits. We included binary variables indicating whether Veterans were prescribed sulfonylureas, meglitinides, metformin, thiazolidinediones, α-glucosidase inhibitors, insulin, or antihypertensive medications (e.g., β-blockers, calcium channel blockers, antihypertensive combinations). ICD-9 and CPT codes were used to create binary indicators for Quan-Elixhauser comorbidities [11] as well as end-stage liver disease, major depression, coronary artery disease, acute myocardial infarction, percutaneous coronary interventions [12], nicotine dependence or smoking cessation, retinopathy, hyperglycemia, lower-limb amputation, and diabetic foot infections [13]. We used CPT codes to create indicators of screenings for retinopathy and ankle-brachial indices [14]. A frailty index ranging from 0 to 1 was also created using 30 variables identified from ICD-9 or CPT codes related to morbidity (e.g., arthritis), functional status (e.g., need for durable medical equipment), cognition and mood (e.g., dementia), sensory impairment (e.g., hearing impairment), or other conditions (e.g., incontinence) [15]. Biomarkers (e.g., BMI, blood pressure, A1C) were calculated as the mean of all measurements during the baseline period Table 2 displays a complete list of included variables and their coded classifications.

**Table 2:** Data dictionary.

Variable description	Variable name	Coding
Age (in years)	AGE	Raw value
Alpha-glucosidase inhibitor prescription	ALPHA	0=No, 1=Yes
Biguanide prescription	BIGUAN	0=No, 1=Yes
Body mass index (BMI)	BMI	Raw value
Diastolic blood pressure	DIASTOLIC	Raw value
Enrollment priority	PRIORITY	1=Priority group 1 (50% of more service-connected disability), 2=Priority group 2 (30–40% service-connected disability), 3=Priority group 3 (10–20% service-connected disability), 4=Priority group 4 (catastrophically disabled/housebound), 5=Priority group 5 (economic hardship), 6=Priority group 6 (no service-connected disability, specific exposures during service, other), 7/8 = Priority groups 7 or 8 (those without service-connected disabilities or economic hardship)
Frailty index	FRAILTY	FRAILTY: The frailty index indicates presence or absence of thirty age-related deficits in health. Deficits were identified using ICD-9 and CPT codes. An individual's frailty was calculated as a ratio of their total health decrements divided by the total possible (30).
HDL cholesterol	HDL	Raw value
Hemoglobin A1c	A1C	Raw value
Inpatient days	N_IP	Count of inpatient days
Insulin prescription	INSULIN	0=No, 1=Yes
LDL cholesterol	LDL	Raw value
Marital status	MARRIED	1=Married, 2=Single, 3=Widowed
Mortality	DEATH_5 DEATH_10	DEATH_5: 0 if alive as of 31 December 2011, 1 otherwise DEATH_10: 0 if alive as of 31 December 2016, 1 otherwise
Other diabetes medication	OTHER_MED	0=No, 1=Yes; part of VA drug class HS5XX excluding drugs in other medication categories
Outpatient visits	N_OP	Count of unique visits
Race	RACE	1=White, 2=Black, 3=Other
Serum albumin	SERUMALB	Raw value
Serum creatinine	SERUMCRE	Raw value
Sex	SEX	0=female, 1=male
Sulfonylurea prescription	SULF	0=No, 1=Yes
Systolic blood pressure	SYSTOLIC	Raw value
Thiazolidinedione prescription	TZD	0=No, 1=Yes
Triglycerides	TRI	Raw value
Urine microalbumin	MICROALB	Raw value

**The following comorbidities were assigned a value of 1 if the relevant diagnosis/procedure codes were present, 0 otherwise:**		

Acute myocardial infarction	AMI	410.x, 411.0, 427.5, 412.x (ICD-9)
AIDS/HIV	HIV	042.x-044.x (ICD-9)
Alcohol abuse	ALCOHOL	265.2, 291.1–291.3, 291.5–291.9, 303.0, 303.9, 305.0, 357.5, 425.5, 535.3, 571.0–571.3, 980.x, V11.3 (ICD-9)
Ankle-brachial index	ABI	93,922, 93,923, 93,924 (CPT)
Blood loss anemia	BLOODLOSS	280 (ICD-9)
Cardiac arrhythmias	ARRHYTHMIA	426.0, 426.13, 426.7, 426.9, 426.10, 426.12, 427.0–427.4, 427.6–427.9, 785.0, 996.01, 996.04, V45.0, V53.3 (ICD-9)
Chronic pulmonary disease	PULMONARY	416.8, 416.9, 490.x-505.x, 506.4, 508.1, 508.8 (ICD-9)
Coagulopathy	COAG	286.x, 287.1, 287.3–287.5 (ICD-9)
Congestive heart failure	CHF	398.91, 402.01, 402.11, 402.91, 404.01, 404.03, 404.11, 404.13, 404.91, 404.93, 425.4–425.9, 428.x (ICD-9)
Coronary arterial disease	CAD	414.01, 414.00, 414.04, 414.03, 414.06, 404.02, 414.05 (ICD-9)
Deficiency anemia	ANEMIA	280.1–280.9, 281.x (ICD-9)
Depression	DEPRESSION	296.2, 296.3, 296.5, 300.4, 309.x, 311 (ICD-9)
Diabetes, complicated	DMCX	250.4–250.9 (ICD-9)
Diabetic foot infections	FEET	040.0, 440.24, 785.4, 730.07, 730.17, 730.27, 730.97, 440.23, 707.14, 707.15, 707.1, 680.7, 682.7, 681.1, 681.10, 681.11 (ICD-9), 75,710,75,716, 75,630, 75,600, 75,605, 75,625, 11,044, 10,060, 10,061, 20,000, 20,005 (CPT)
Drug abuse	DRUGS	292.x, 304.x, 305.2–305.9, V65.42 (ICD-9)
End-stage liver disease for cirrhosis or alcoholic fatty liver	ESLD	571.2, 571.5, 571.6, 571.0, 571.1, 572.2, 572.3, 572.4 (ICD-9)
Fluid & electrolyte disorders	FLUIDSLYTES	253.6, 276.x (ICD-9)
Hyperglycemia	HYPERG	250.3, 250.8, 251.0, 251.1, 251.2, 270.3, 775.0, 775.6, 962.3 (ICD-9)
Hypertension, complicated	HTNCX	402.x-405.x (ICD-9)
Hypertension, uncomplicated	HTN	401.x (ICD-9)
Hypothyroidism	HYPOTHYROID	240.9, 243.x, 244.x, 246.1, 246.8 (ICD-9)
Liver disease	LIVER	070.22, 070.23, 070.32, 070.33, 070.44, 070.54, 070.6, 070.9, 456.0–456.2, 570.x, 571.x, 572.2–572.8, 573.3, 573.4, 573.8,573.9, V42.7 (ICD-9)
Lower limb amputation	AMPUTATION	V49.71-V49.77, V52.1 (ICD-9), 27,888, 28,800–28,805, 27,290, 27,598, 27,880–27,886, 27,590–27,592, 27,290–27,295,27,594–27,596, 26,910, 28,810–28,825 (CPT)
Lymphoma	LYMPHOMA	200.x-202.x, 203.0, 238.6 (ICD-9)
Metastatic cancer	METS	196.x-199.x (ICD-9)
Obesity	OBESITY	278 (ICD-9)
Other neurological disorders	NEUROOTHER	331.9, 332.0, 332.1, 333.4, 333.5, 333.92, 334.x335.x, 336.2, 340.x, 341.x, 345.x, 348.1, 348.3, 780.3, 784.3 (ICD-9)
Paralysis	PARALYSIS	334.1, 342.x, 343.x, 344.0- 344.6, 344.9 (ICD-9)
Peptic ulcer disease excluding bleeding	PUD	531.7, 531.9, 532.7, 532.9, 533.7, 533.9, 534.7, 534.9 (ICD-9)
Percutaneous coronary interventions	PCI	92,920, 9291, 92,924, 92,925, 92,928, 92,929, 92,933, 92,934, 92,937, 92,938, 92,941, 92,943, 92,944, 92,973, 92,980, 92,981, 92,982, 92,984, 92,995, 92,996 (CPT)
Peripheral vascular disorders	PVD	093.0, 437.3, 440.x, 441.x, 443.1–443.9, 447.1, 557.1 557.9, V43.4 (ICD-9)
Psychoses	PSYCHOSES	293.8, 295.x, 296.04, 296.14, 296.44, 296.54, 297.x, 298.x (ICD-9)
Pulmonary circulation disorders	PHTN	415.0, 415.1, 416.x, 417.0, 417.8, 417.9 (ICD-9)
Renal failure	RENAL	403.01, 403.11, 403.91, 404.02, 404.03, 404.12, 404.13, 404.92, 404.93, 585.x, 586.x, 588.0, V42.0, V45.1, V56.x (ICD-9)
Retinopathy	RETINOPATHY	250.5x, 362.0, 379.23, 362.01–362.07 (ICD-9)
Retinopathy screening	RETSCREEN	92,250, 99,243, 92,227, 92,228 (CPT)
Rheumatoid arthritis/collagen vascular diseases	RHEUMATIC	446.x, 701.0, 710.0–710.4, 710.8, 710.9, 711.2, 714.x, 719.3, 720.x, 725.x, 728.5, 728.89, 729.30 (ICD-9)
Severe depression	SEVERE_DEP	296.3, 298.0, 300.4 (ICD-9)
Smoking	SMOKER	305.01, 649.x, 989.84, V15.82 (ICD-9), 99,406, 99,407, S9075, S9453, G0436, G0437 (CPT)
Solid tumor without metastasis	TUMOR	140.x-172.x, 174.x-195.x (ICD-9)
Valvular disease	VALVULAR	093.2, 394.x-397.x, 424.x, 746.3–746.6, V42.2, V43.3 (ICD-9)
Weight loss	WEIGHTLOSS	260.x-263.x, 783.2, 799.4 (ICD-9)

For completeness and reproducibility, we have included SQL scripts that were used to extract raw data from the VHA CDW, an R script to prepare the analytic file, and the R script used to estimate the mortality risk prediction models. Pre-processed, deidentified data files are also available in CSV, Stata (.dta), and R (.rds) formats.

We note several limitations with these data. First, our determination of diabetes status may include a small amount of misclassification. The criteria we used to identify patients with diabetes based on VHA electronic health records have been previously validated, achieving high sensitivity (93%) and specificity (98%) compared to patients’ self-reported health status [8]. Second, we obtained administrative data from both VHA and Medicare, but our data do not capture health services utilization from other payers. Lastly, while the CDW incorporates death records from several federal sources, a small number of Veteran deaths may remain unreported.

### File inventory

• SQL scripts to extract data from the VHA CDW.
• R statistical code to pre-process the data.
• R statistical code to estimate mortality risk prediction models.
• Deidentified individual-level datasets of mortality and patient characteristics (processed).

## Ethics Statement

The study was reviewed and approved by the VA Boston Health Care System's Institutional Review Board (protocol #1584905-2). A waiver of informed consent was granted for this database-only study, with identifiable information limited to the minimum required to complete the study. Contacting patients to provide informed consent, in addition to being infeasible due to sample size, would thus increase the risks associated with a breach of confidentiality. Release of deidentified datasets was also authorized as part of this publication.

The Privacy Office of the Veterans Affairs Boston Healthcare System have certified these datasets are de-identified and may be publicly released as part of this publication.

## Data Availability

A Novel Dataset of Predictors of Mortality for Older Veterans Living with Type II Diabetes (Original data) (Mendeley Data).

## CRediT authorship contribution statement

**Avi U. Vaidya:** Writing – review & editing. **Gabriel A. Benavidez:** Writing – review & editing. **Julia C. Prentice:** Funding acquisition, Conceptualization, Writing – review & editing. **David C. Mohr:** Data curation, Writing – review & editing. **Paul R. Conlin:** Funding acquisition, Conceptualization, Writing – review & editing. **Kevin N. Griffith:** Writing – review & editing, Conceptualization, Data curation.

## Declaration of Competing Interest

David Mohr, Paul Conlin, and Kevin Griffith are investigators at the VA Boston Healthcare System. The content is solely the responsibility of the authors and does not necessarily represent the views of the VHA, which did not have editorial input or control over this research. Thus, the views expressed in this article are those of the authors and do not necessarily reflect the position or policy of the Department of Veterans Affairs or the United States government.
